# Hypothermic stunning of green sea turtles in a western Gulf of Mexico foraging habitat

**DOI:** 10.1371/journal.pone.0173920

**Published:** 2017-03-17

**Authors:** Donna J. Shaver, Philippe E. Tissot, Mary M. Streich, Jennifer Shelby Walker, Cynthia Rubio, Anthony F. Amos, Jeffrey A. George, Michelle R. Pasawicz

**Affiliations:** 1 National Park Service, Padre Island National Seashore, Corpus Christi, Texas, United States of America; 2 Conrad Blucher Institute, Texas A&M University–Corpus Christi, Corpus Christi, Texas, United States of America; 3 Marine Science Institute, University of Texas at Austin, Port Aransas, Texas, United States of America; 4 Sea Turtle, Inc., South Padre Island, Texas, United States of America; 5 Florida Fish and Wildlife Commission, Tallahassee, Florida, United States of America; Deakin University, AUSTRALIA

## Abstract

Texas waters provide one of the most important developmental and foraging habitats for juvenile green turtles (*Chelonia mydas*) in the western Gulf of Mexico, but hypothermic stunning is a significant threat and was the largest cause of green turtle strandings in Texas from 1980 through 2015; of the 8,107 green turtles found stranded, 4,529 (55.9%) were victims of hypothermic stunning. Additionally, during this time, 203 hypothermic stunned green turtles were found incidentally captured due to power plant water intake entrapment. Overall, 63.9% of 4,529 hypothermic stunned turtles were found alive, and 92.0% of those survived rehabilitation and were released. Numbers of green turtles recorded as stranded and as affected by hypothermic stunning increased over time, and were most numerous from 2007 through 2015. Large hypothermic stunning events (with more than 450 turtles documented) occurred during the winters of 2009–2010, 2010–2011, 2013–2014, and 2014–2015. Hypothermic stunning was documented between November and March, but peaked at various times depending on passage of severe weather systems. Hypothermic stunning occurred state-wide, but was most prevalent in South Texas, particularly the Laguna Madre. In the Laguna Madre, hypothermic stunning was associated with an abrupt drop in water temperatures strong northerly winds, and a threshold mean water temperature of 8.0°C predicted large turtle hypothermic stunning events. Knowledge of environmental parameters contributing to hypothermic stunning and the temporal and spatial distribution of turtles affected in the past, can aid with formulation of proactive, targeted search and rescue efforts that can ultimately save the lives of many affected individuals, and aid with recovery efforts for this bi-national stock. Such rescue efforts are required under the U.S. Endangered Species Act and respond to humanitarian concerns of the public.

## Introduction

Hypothermic stunning occurs when sea turtles are exposed to water temperatures that drop below about 10°C [1,2]. During these times, turtles lose their ability to swim and dive, float to the surface, and can succumb between 5°C and 6°C if not rescued [2–6]. Sea turtles feeding in shallow enclosed areas are the most susceptible to hypothermic stunning [1,7,8]. Sudden drops in water temperature due to excessively cold weather fronts have resulted in mortality to sea turtles on the Texas coast, particularly in the very shallow waters of the Laguna Madre [9–11]. Hypothermic stunning of sea turtles during cold weather episodes has also been reported in Western Europe [3,12], Hawaii [13], elsewhere along the U.S. Atlantic Ocean and Gulf of Mexico coasts [14–25], and Gulf of Mexico coast in Tamaulipas, Mexico (Peña, pers. comm.). Species affected have included green (*Chelonia mydas*), Kemp's ridley (*Lepidochelys kempii*), loggerhead (*Caretta caretta*), and hawksbill (*Eretmochelys imbricata*) turtles.

The green turtle is listed under the U.S. Endangered Species Act of 1973 as threatened throughout its range with the exception of the distinct population segments in the central south Pacific, central west Pacific, and Mediterranean, which are afforded endangered status. Green turtles were once so abundant in Texas waters that they were commercially exploited [9,10,26–28]. The green turtle fishery began in Texas during the mid-1800s and was in excess of 230,000 kg/year at its peak, but catch declined to such an extent that this fishery was virtually eliminated by 1900, and was extremely low when harvest was outlawed in 1963 [9,10]. There is evidence that over-harvesting and hypothermic stunning during the severe freezes of 1894–1895 and 1899 decimated the population [29]. Prior to the passage of the U.S. Endangered Species Act, rescue and rehabilitation efforts were not undertaken, and thus most turtles affected by hypothermic stunning likely died or were taken for consumption [9,10,27–29]. However, after decades of protection under the U.S. Endangered Species Act, the aggregation of green turtles inhabiting Texas waters has increased and Texas waters now serve as one of the most important developmental and foraging habitats for this species in the western Gulf of Mexico [11,30,31].

Green turtles migrate extensively and occur in different habitats during different stages of their lives. For their first year of life or longer, green turtles are pelagic and passively drift in oceanic currents and gyre systems [32–34]. At roughly 20–40 cm curved carapace length or larger, juveniles leave the pelagic zone and actively recruit to shallow water developmental habitats in tropical and temperate zones [11,15,16,35–37], where they feed primarily on seagrasses and macroalgae [22,38–41]. Juveniles may enter a series of developmental habitats to locate foraging resources and appropriate thermal conditions [32,33,37,42,43]. Generally, pubescent green turtles move into adult foraging habitats which may coincide with juvenile developmental areas and adults migrate between foraging and nesting grounds [44–46].

**Fig 1:**
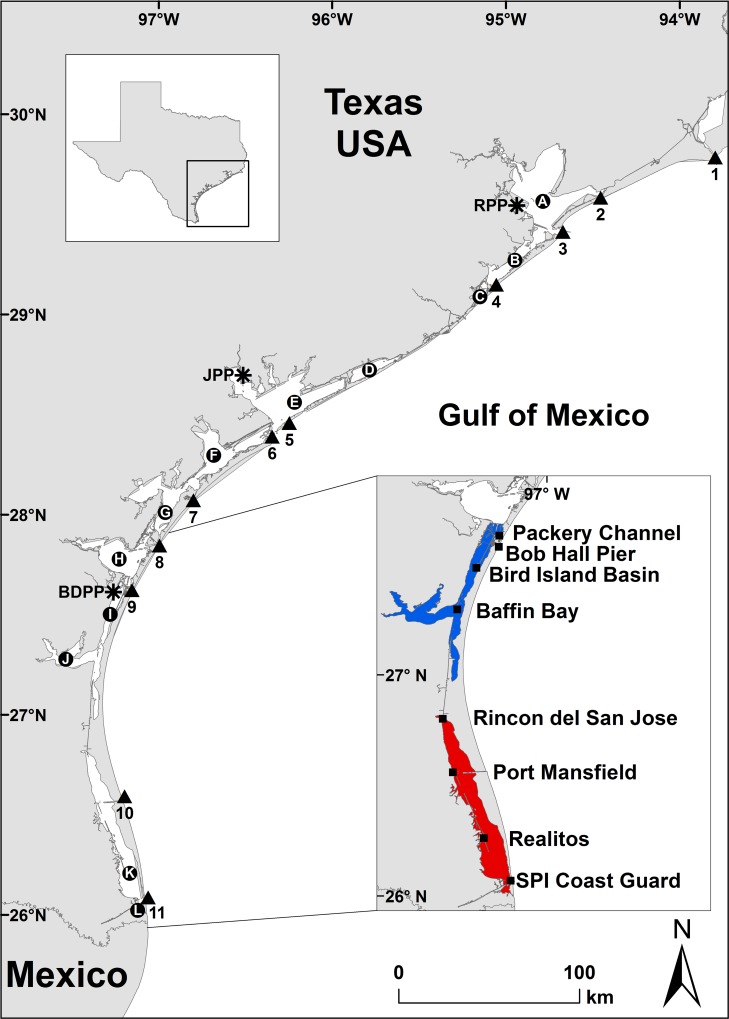
Map of Texas coast with numbers representing the 12 lettered bays systems (circles), 11 numbered channels and passes (triangles), 3 power plants (asterisks), and insert map of 8 National Water Level Observation Network (NWLON) and Texas Coastal Ocean Observation Network (TCOON) environmental monitoring stations (squares) along the Upper Laguna Madre (blue) and Lower Laguna Madre (red) of South Texas. The 12 bays are arranged from north to south: A = Galveston Bay, B = West Bay, C = Christmas Bay, D = East Matagorda Bay, E = Matagorda Bay, F = San Luis Bay, G = Aransas Bay, H = Corpus Christi Bay, I = Upper Laguna Madre, J = Baffin Bay, K = Lower Laguna Madre, and L = South Bay. The 11 channels and passes are arranged from north to south: 1 = Sabine Pass, 2 = Rollover Pass, 3 = Bolivar Roads Channel, 4 = San Luis Pass, 5 = Matagorda Ship Channel, 6 = Pass Cavallo, 7 = Cedar Bayou Pass, 8 = Aransas Pass Ship Channel, 9 = Packery Channel, 10 = Mansfield Channel, and 11 = Brazos Santiago Pass. The 3 power plants are arranged from north to south: RPP = PH Robinson Power Plant, JPP = Joslin Power Plant, and BDPP = Barney Davis Power Plant. The eight stations are arranged from north to south: Packery Channel, Bob Hall Pier (NWLON), Bird Island Basin, Baffin Bay, Rincon del San Jose, Port Mansfield, Realitos and South Padre Island (SPI) Coast Guard. (TIF)

The Texas coast consists of barrier islands with dynamic sandy beaches and high energy nearshore Gulf of Mexico waters [47]. Seven major bays (Sabine Lake, Galveston, Matagorda, San Antonio, Aransas, Corpus Christi, and the Laguna Madre) and several smaller bays are located between these barrier islands and mainland Texas (Fig 1). Seagrass beds have a patchy distribution among Texas bays [48] and eighty percent of seagrass beds in Texas occur in the 209 km long hypersaline Laguna Madre [49–51]. Multiple navigable passes and channels connect the bays with the Gulf of Mexico (Fig 1). Many of these channel entrances are reinforced with jetties constructed of large granite rocks. Macroalgae species grow on the granite rocks and on the other hard structures such as fishing piers, docks, oil and gas platforms, bridge support structures, and natural reefs that occur in the bays and nearshore Gulf of Mexico waters [52–57].

Nearly all green turtles in Texas waters are juveniles [11,31,58]. Few green turtles nest in Texas [11] and the predominant natal origin of the Texas foraging aggregation is likely the Gulf coast of Mexico and to a lesser extent in the Caribbean and Florida [59]. In Texas, juveniles use bays as developmental habitat and passes as intermediate developmental habitat between the pelagic habitat and lagoonal feeding pastures and as a route for movement between the Gulf of Mexico and these bays [11,30,36,60]. In fact, based on documentation of large numbers of individuals stranded and captured during netting studies, and individuals monitored via radio, sonic, and satellite telemetry [11,30,31,36,60–62], the Laguna Madre, Mansfield Channel, and Brazos Santiago Pass in South Texas are likely among the most important developmental habitats for green turtles in the western Gulf of Mexico. However, the relatively shallow waters of the Laguna Madre and limited number of passes between it and the Gulf of Mexico can serve to entrap these turtles during periods of very cold temperatures, resulting in hypothermic stunning.

Stranded sea turtles are dead or live turtles that are found washed ashore or floating; live floating turtles are generally in a weakened condition [63]. Several factors have been implicated in sea turtle strandings along the Texas coast [58,64], but hypothermic stunning was the largest cause of green turtles strandings documented there by the Sea Turtle Stranding and Salvage Network (STSSN) [65] (http://www.sefsc.noaa.gov/species/turtles/strandings.htm) from 1980 through 1996 [11]. More information is needed to quantify the recent magnitude of hypothermic stunning in Texas, temporal and spatial distribution of turtles affected, and environmental parameters contributing to hypothermic stunning there. These findings could be used to help formulate proactive, targeted search and rescue efforts to reduce the number of green turtles killed by hypothermic stunning. Such actions would aid with restoration of the green turtle population in Texas, which is in the recovery phase after being depleted more than a century ago. The genetic isolation of this population highlights the significance of Texas as developmental habitat and reiterates the importance of continued international cooperation to facilitate recovery of this stock [59]. Rescue and rehabilitation efforts are required under the U.S. Endangered Species Act and respond to humanitarian concerns of the public, but more information is needed to quantify the success rate of rehabilitation and contribution of repatriated turtles to population recovery [66,67].

Our objectives were to: 1. Determine the numbers of green turtles affected by hypothermic stunning and relative contribution of hypothermic stunning to strandings of green turtles in Texas since 1980; 2. Determine the spatial distribution and temporal trends in the numbers of green turtles recorded hypothermic stunned in Texas; 3. Determine the success rate of rescue and rehabilitation for hypothermic stunned green turtles in Texas; and, 4. Determine the relationship of water temperature and other oceanic and atmospheric parameters to the onset and variations in green turtle hypothermic stunning in the Laguna Madre. These analyses will address information needs and recovery task priority actions for green turtles in the western Gulf of Mexico [68–70].

## Methods

### Detection and documentation of stranded and incidentally captured turtles

The STSSN documented sea turtles found stranded and incidentally captured (i.e., entrapped in intake canals of power plants or captured during active commercial or recreational fishing) in Texas from 1980 through 2015. Turtles were located in response to information provided by the public or personnel from other organizations, or during systematic surveys conducted by STSSN participants. Each turtle was documented on a standardized STSSN form that was forwarded to the state and subsequently to the national STSSN coordinators. Those turtles documented due to hypothermic stunning were identified. Records were categorized as from offshore (beaches or waters of the Gulf of Mexico) and inshore (beaches or waters of passes and bays) areas.

Live hypothermic stunned turtles were taken to rehabilitation facilities located in Texas, and held there for periods ranging from less than a week to up to one year, depending on the condition of the turtle. Most that survived rehabilitation were marked with Inconel tags (National Band and Tag Company, Newport, Kentucky, U.S.) applied externally on one or two flippers and a Passive Integrated Transponder (PIT) tag (Biomark, Boise, Idaho, U.S.) implanted into a fore-flipper [71]. Turtles were released when water temperatures warmed. From 1980 through 2009, most were returned to waters near where they had been found, but from 2010 onward most were released on South Texas Gulf beaches. During these later years, the large numbers of turtles found exceeded capacity to hold them for prolonged periods of rehabilitation; Gulf releases reduced holding times since warmer waters were available there than in the bays.

### Permitting and animal welfare

This study was carried out in strict accordance with the Guide for the Care and Use of Laboratory Animals of the National Institutes of Health. Work by National Park Service personnel was authorized under USFWS Permit TE840727-3, TPWD Scientific Permit SPR-0190-122, and NPS Institutional Animal Care Protocols NPS IACUC 2011–15.

### Data analysis of turtles

The Texas STSSN database was queried for records of sea turtles found stranded in Texas from 1980 through 2015. The STSSN database was also queried for records of hypothermic stunned turtles that had been incidentally captured on the intake screens at the Barney Davis Power Plant (BDPP) in Corpus Christi [72], P.H. Robinson Power Plant in Galveston, or Joslin Power Plant in Point Comfort (Fig 1). Both stranded and incidentally captured hypothermic stunned turtles were included in analyses of temporal and spatial distributions of hypothermic stunned turtles. However, incidentally captured turtles were excluded from analyses restricted to stranded individuals, since they are not considered true strandings [63].

We used ArcGIS 10.2.1 [73] to display the distribution of hypothermic stunned green turtles from 1980–2015 within 5.5 km horizontal bands (0.5 degrees of latitude) from north south in the study area. We then calculated the density of hypothermic stunned turtles per km^2^ across the study area using the Point Density Analysis Tool.

For statistical analyses, winters (November through March), were designated by the year that they began, and weeks of the hypothermic stunning season (mid-November through mid-March) were numbered consecutively from 1 through 17.

All means are followed by ± one standard deviation. Data were tested for normality and homogeneity of variance prior to using parametric procedures. When parametric assumptions were not met, equivalent non-parametric procedures were used. Pearson Product Moment Correlation was used to examine the relationship between the: 1) duration of each hypothermic stunning season (number of days from first to last hypothermic stunning during a winter) and numbers of hypothermic stunned turtles found, 2) duration of the hypothermic stunning season and percent of hypothermic stunned turtles found alive, and 3) numbers of hypothermic stunned turtles found and percent that were found alive.

### Data analysis of environmental parameters

We examined the relationship of water temperature, air temperature, wind speed, wind gust, wind direction, barometric pressure, water level, and water level standard deviation (wave proxy) [74] to variations in green turtle hypothermic stunning in and near the Laguna Madre. The Laguna Madre has a mean depth of 1.2 m, which is less than any other Texas estuary [75]. Circulation of seawater into and out of the Laguna Madre is highly restricted. There are three direct water exchange passages to the Gulf of Mexico including the Brazos Santiago Pass, Mansfield Channel, and Packery Channel which was dredged in 2005 and appears to have facilitated usage of the Upper Laguna Madre by juvenile green turtles. Environmental data were acquired from the Texas Coastal Ocean Observation Network (TCOON) and the National Water Level Observation Network (NWLON) (Fig 1). Hypothermic stunned green turtles found in the Laguna Madre, three adjoining passes, and three nearby Gulf of Mexico beach segments within 1.61 km north and south of those passes, and incidentally captured at the BDPP were included in the analyses. Turtles found within the adjoining bays (i.e., Corpus Christi Bay and Baffin Bay) were excluded. Environmental conditions were then extracted from a nearby TCOON or NWLON station for each of the 3,381 hypothermic stunned turtle cases based on their stranding or incidental capture locations and timing, resulting in 345 hypothermic stunning days with associated environmental data. Measurements from Bob Hall Pier were used for the three Gulf of Mexico beach segments. Cases with missing or insufficient measurements to compute a daily mean were not imputed and left out of statistical computations.

Our environmental data analysis was restricted to turtles found during the winters of 1995–1996 through 2014–2015, to match environmental parameter data availability. Part of the analysis was conducted by associating the 345 individual hypothermic stunning days with environmental parameter data collected at the nearest station. Further analysis was based on associating all hypothermic stunned turtle cases with environmental parameter measurements at the Upper Laguna Madre station (Bird Island Basin), further reducing the data set to 220 hypothermic stunning days. This station provided the most complete time series over the study period with data availability of 96% for water temperatures and wind, and 98% for water levels. Data availability for other stations depended substantially on location and was not considered as reliable for robust long term averages. Using Upper Laguna Madre environmental parameters to characterize the full study area was also possible due to the strong correlations between that station and the seven other stations: 0.90 and higher for water temperature, 0.97 and stronger for air temperature, and at least 0.96 for barometric pressure over the respective time spans with measurements available at pairs of stations (S1 Table). Correlation strengths were lower for the other variables such as water levels and wind measurements, with values generally decreasing with distance from the Bird Island Basin station. A daily breakdown was selected from 1800 h to 1800 h the next day to match the documentation time of hypothermic stunned turtles (i.e., up to 1800 h in the afternoons); 1800 h CST also corresponds to 0000 h UTC the next day outside of daylight savings time and hence UTC days were selected as the unit daily period. One-day mean time series were created from hourly measurements for each variable. To be included in the data set, there could be no more than two missing hourly measurements. The start of our data set corresponds to 1 November 1995 0000 h UTC, and the time span considered 31 October 1995 1800 h (1 November 1995 0000 h UTC) through 1 April 2015 1700 h CST (1 April 2015 2300 h UTC). To examine near real-time impacts of these variables, only turtles that were condition code 0 (alive) or 1 (fresh dead) were included.

Finally, we analyzed monthly mean water temperatures in the Laguna Madre in the context of broad sea surface temperature pattern fluctuations. If significant, such relationships could be helpful for operational preparation ahead of the winter. Monthly Upper Laguna Madre water temperatures at the Bird Island Basin station were seasonally detrended and correlated with three climatic indexes: the El Nino Southern Oscillation (ENSO), the Atlantic Multi-Decadal Oscillation (AMO), and the Pacific Decadal Oscillation (PDO). Monthly index time series were obtained from the following sources: ENSO (ONI Index) (http://www.cpc.ncep.noaa.gov/products/analysis_monitoring/ensostuff/ensoyears.shtml), AMO (http://www.esrl.noaa.gov/psd/data/timeseries/AMO/), and PDO (http://research.jisao.washington.edu/pdo/PDO.latest). Pearson Pair Correlation Coefficients and their respective p-values were first computed for all monthly values without temporal lags and then only for the winter (November–March) monthly means. Correlations with the climatic index time series were then estimated with temporal lags ranging from one month to 30 months.

## Results

### Turtle analysis

**Fig 2:**
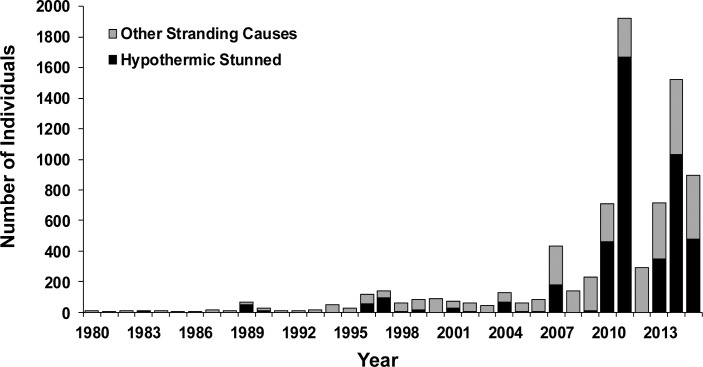
Annual number of green turtles found stranded (including post-hatchlings) in Texas, 1980 through 2015.

Of the 8,107 green turtles found stranded in Texas from 1980 through 2015, 4,529 (55.9%) stranded due to hypothermic stunning and 3,578 (44.1%) stranded due to other causes. The annual numbers of green turtles found stranded increased exponentially from 1980 through 2015 (r^2^ = 0.6145, p < 0.0001) (Fig 2). Annual numbers of green turtles found stranded, found stranded due to hypothermic stunning, and found stranded due to other factors (Fig 2), and annual percentages of green turtles found stranded due to hypothermic stunning were significantly larger during 2007 through 2015 than during 1980 through 2006 (in all cases: Mann-Whitney U = 0.000–65.500, p < 0.05).

From 1980 through 2015, 4,732 hypothermic stunned green turtles were recorded in Texas, including the 4,529 stranded individuals and 203 individuals found incidentally captured at the BDPP (n = 200), P.H. Robinson Power Plant (n = 2), and Joslin Power Plant (n = 1). The largest hypothermic stunning totals (strandings and incidental captures) were during the winters of 2009–2010, 2010–2011, 2013–2014, and 2014–2015, with more than 450 green turtles recorded during each of those winters (Table 1). In addition to 4,732 green turtles, 12 loggerhead, 11 hawksbill, six Kemp’s ridley, and seven turtles for which species could not be identified were also found hypothermic stunned through incidental capture at the BDPP or stranding in South Texas from 1980 through 2015.

**Table 1:** Number of hypothermic stunned green turtles (*Chelonia mydas*) recorded in Texas, 1980 through 2015.

Winter	Date Start	Date End	Total Number of Days	Total Number Found	Number Found Inshore	Number Found Offshore	Percent Found Alive
1981–1982	1/11/1982	1/13/1982	3	2	2	0	100.0
1983–1984	12/18/1983	1/13/1984	27	5	5	0	80.0
1988–1989	2/6/1989	2/14/1989	9	46	46	0	34.8
1989–1990	12/20/1989	2/14/1990	57	14	13	1	7.1
1990–1991	12/27/1990	12/30/1990	4	2	1	1	0.0
1995–1996	12/23/1995	2/25/1996	106	58	56	2	89.7
1996–1997	12/20/1996	2/7/1997	50	101	95	6	42.6
1997–1998	12/15/1997	12/15/1997	1	1	1	0	100.0
1998–1999	12/13/1998	1/19/1999	38	19	19	0	21.1
1999–2000	12/3/1999	2/1/2000	61	8	4	4	75.0
2000–2001	11/21/2000	2/11/2001	83	50	49	1	74.0
2001–2002	12/1/2001	1/7/2002	38	8	6	2	87.5
2004–2005	12/25/2004	1/5/2005	12	72	68	4	41.7
2005–2006	12/5/2005	2/21/2006	79	7	4	3	85.7
2006–2007	12/6/2006	2/9/2007	66	180	163	17	68.9
2009–2010	12/5/2009	2/27/2010	85	466	453	13	35.8
2010–2011	12/26/2010	2/25/2011	62	1670	1632	38	64.3
2012–2013	12/20/2012	1/29/2013	41	48	44	4	62.5
2013–2014	11/25/2013	3/7/2014	103	1256	1227	29	74.0
2014–2015	11/13/2014	3/6/2015	114	719	675	44	68.9
Total				4732	4563	169	63.9

**Fig 3:**
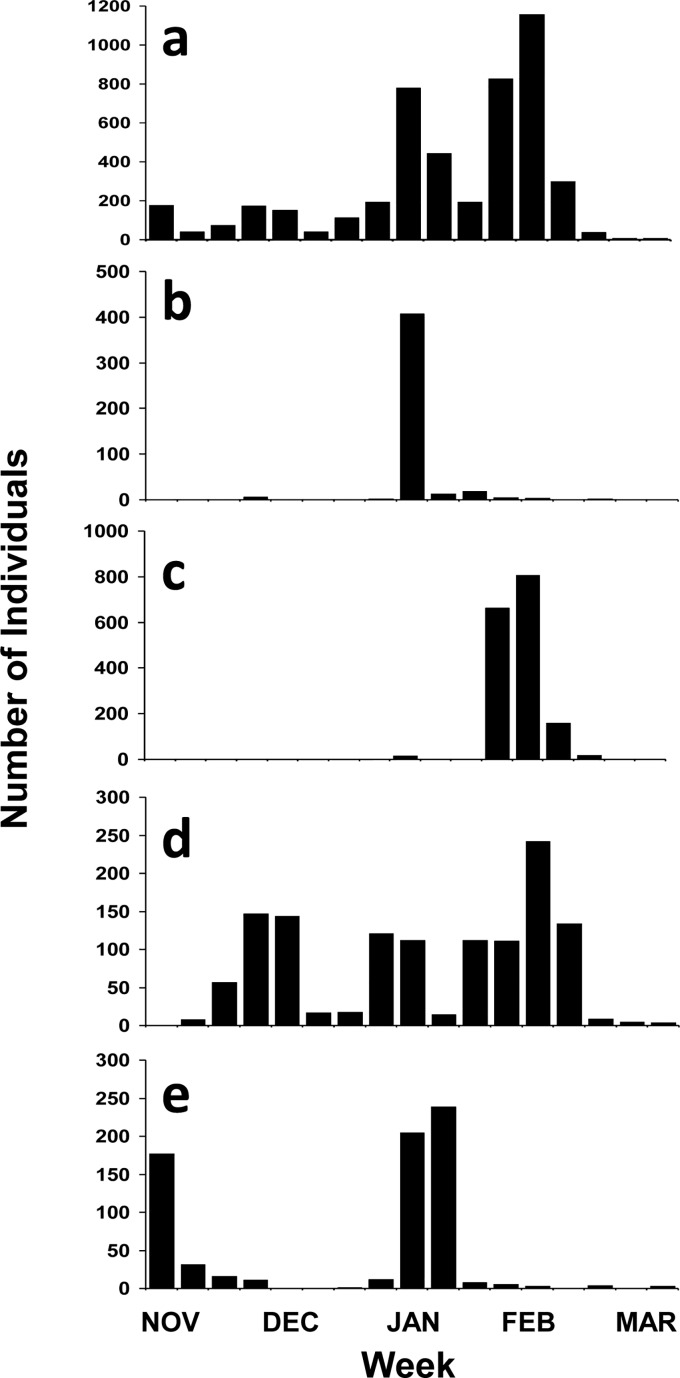
Weekly number of hypothermic stunned green turtles (strandings and incidental captures) recorded in Texas. Includes those recorded during (a) all years combined (1980–2015) and during the winters of (b) 2009–2010, (c) 2010–2011, (d) 2013–2014, and (e) 2014–2015.

Hypothermic stunning was documented between November and March, but peaked at various times during different winters and sometimes multiple times during a winter (Fig 3), depending upon the timing of passage of severe weather systems. The duration of each hypothermic stunning season ranged from 1 to 114 days (mean = 49.9 ± 33.7 days, n = 20). The duration of the hypothermic stunning season and the numbers of hypothermic stunned turtles found were positively correlated (r^2^ = 0.506, p < 0.05).

**Fig 4:**
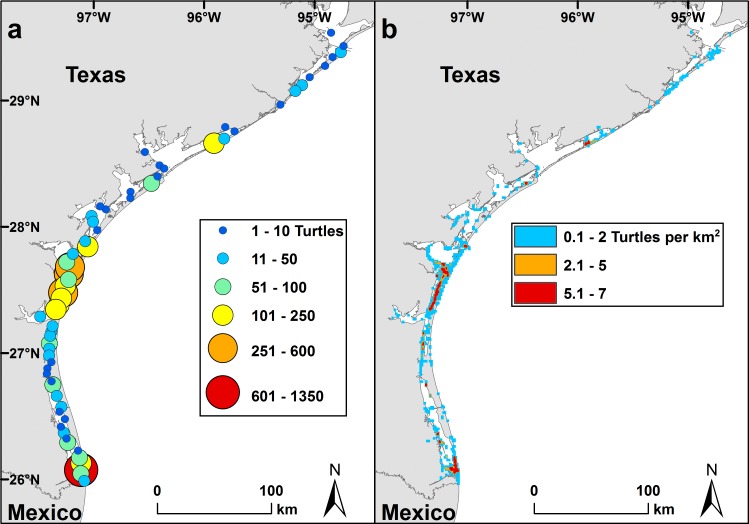
Distribution of hypothermic stunned green turtles (strandings and incidental captures) recorded in Texas from 1980 through 2015. Data displayed as (a) number of turtles within 5.5 km horizontal bands (0.5 degrees of latitude) from north to south and (b) density (number of turtles per km^2^) across the study area.

Of the 4,732 hypothermic stunned green turtles, 4,563 (96.4%) were found inshore and 169 (3.6%) were found offshore. Hypothermic stunning occurred state-wide, but 4,343 (91.8%) of those documented were found in South Texas (latitude < 27.901, Aransas Pass southward) (Fig 4).

Of the 4,732 hypothermic stunned green turtles recorded, 3,026 (63.9%) were found alive and 1,706 (36.1%) were dead when located (Table 1). The annual percent of hypothermic stunned turtles found alive was not correlated with the total number of hypothermic stunned turtles found (r^2^ = 0.061, p > 0.05) or the duration of the hypothermic stunning season (r^2^ = 0.128, p > 0.05). Early in the event most turtles were found alive, whereas the numbers located dead increased the longer that the event persisted.

Of the 2,817 hypothermic stunned turtles found alive during the winters of 2006–2007 through 2014–2015, 203 died during rehabilitation or were euthanized, 12 were retained in captivity, 10 were of unknown fate, and 2,592 survived rehabilitation and were released when water temperatures increased, for a rehabilitation success rate of 92.0% (Table 2). Of the 2,592 turtles released after rehabilitation, 75 were subsequently recorded stranded (i.e., recaptured) due to hypothermic stunning (n = 47) or another cause (n = 28), for a tag return rate of 2.9%. Of the 75 recaptured, 82.7% (n = 62) were found alive and 17.3% (n = 13) were found dead (Table 3). Only 15 of the 75 recaptured turtles were captured and recaptured within the same winter, and of those, four were recaptured due to hypothermic stunning. Most were initially captured inshore (98.7%, n = 74), released offshore (86.3%, n = 64), and subsequently recaptured inshore (73.4%, n = 55). All recaptures were within Texas except one individual found hypothermic stunned in the Upper Laguna Madre, rehabilitated, and released in the Upper Laguna Madre, and recaptured alive in Veracruz, Mexico 205 days after release.

**Table 2:** Rehabilitation success rates for live green turtles (*Chelonia mydas*) found hypothermic stunned in Texas during the winters of 2006–2007 through 2014–2015. Turtles that subsequently stranded with tags were recorded as recaptured.

Winter	Total Number of Turtles	Number Released^1^	Number Died^2^	Number Captive^3^	Number Unknown^4^	Rehab. Success Rate^5^	Number Recaptured	Recapture Rate^6^
2006–2007	124	108	15	0	1	0.871	4	0.037
2009–2010	167	146	15	0	6	0.874	16	0.110
2010–2011	1073	1003	70	0	0	0.935	25	0.025
2012–2013	30	29	0	1	0	0.967	3	0.103
2013–2014	928	853	69	3	3	0.919	24	0.028
2014–2015	495	453	34	8	0	0.915	3	0.007
Total	2817	2592	203	12	10	0.920	75	0.029

^1^ Successfully rehabilitated and released.

^2^ Died during rehabilitation or euthanized.

^3^ Remains in captivity.

^4^ Unknown whether died, was released, or remains in captivity.

^5^ Rehabilitation success rate = number released divided by total number of turtles.

^6^ Recapture rate = number recaptured divided by number released.

Additionally, of the 4,339 hypothermic stunned turtles that were found dead or alive during the winters of 2006–2007 through 2014–2015, 19 were recaptures of turtles that had previously stranded due to other causes. Of these 19 recaptures of previously tagged turtles, 17 were found alive and two were found dead (Table 3). The majority were initially captured offshore (63.2%, n = 12), released inshore (89.5%, n = 17), and subsequently recaptured inshore (94.7%, n = 18).

**Table 3:** Duration (days at large) and distance (km) between release and recapture for live rehabilitated green turtles (*Chelonia mydas*) found hypothermic stunned in Texas during the winters of 2006–2007 through 2014–2015. Includes a) turtles initially recorded as hypothermic stunned, rehabilitated and released, and recaptured due to hypothermic stunning or another cause; and b) turtles initially recorded as stranded due to another cause, rehabilitated and released, and recaptured due to hypothermic stunning.

	Duration (days)		Distance (km)		
Recapture Condition	Mean ± SD	Range	Mean ± SD	Range	N
a) Initial hypothermic stunned					
Found alive	502.8 ± 482.9	3–1446	52.7 ± 124.6	1.9–923.2	62
Found dead	391.8 ± 292.9	10–1034	51.6 ± 63.8	7.0–193.7	13
Total	483.5 ± 456.0	3–1446	52.5 ± 116.0	1.9–923.2	74
b) Initial other cause					
Found alive	558.9 ± 461.9	29–1405	63.7 ± 80.0	0.1–197.9	17
Found dead	184.0 ± 193.7	47–321	32.7 ± 5.0	29.2–36.3	2
Total	519.5 ± 453.5	29–1405	60.5 ± 76.1	0.1–197.9	19

### Environmental parameter analysis

**Fig 5:**
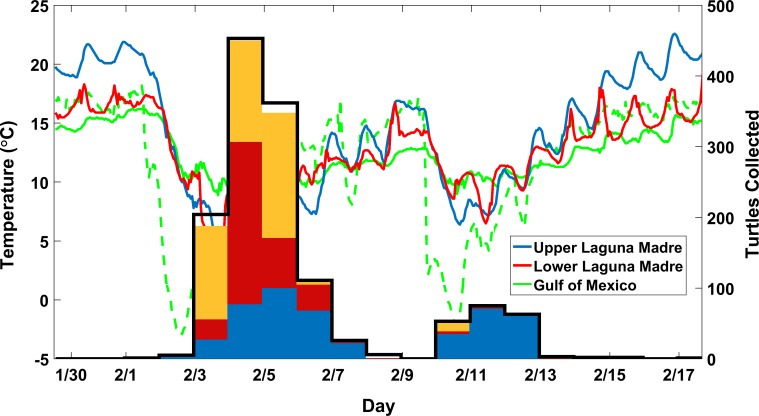
Water temperatures at different locations of the study area (full lines) and air temperature at Bob Hall Pier (dashed line) during the period 30 January to 17 February 2011 (UTC), including two of the largest hypothermic stunning events (1–8 February and 10–17 February) of the study period. Water and air temperatures two days prior to the first event were included for comparison. The bars indicate the chronology of the hypothermic stunned turtles recorded in the Upper Laguna Madre (blue), Lower Laguna Madre (red), Brazos Santiago Pass (orange) and rest of the study area (white).

The environmental parameter analysis included green turtles found in condition codes 0 and 1, in the Laguna Madre, three adjoining passes, and nearby Gulf of Mexico beachfront (Fig 1), during the winters of 1995–1996 through 2014–2015. These 3,381 turtles included 3,190 stranded and 191 incidentally captured, of which 3,346 were found inshore and 35 offshore near the passes. Environmental conditions were associated with the 3,381 cases. The vast majority of turtles (99%) were found during or shortly after the passage of strong cold fronts. The largest hypothermic stunning event took place on 1–8 February 2011, with a total of 1,168 turtles recorded, followed shortly by a second event on 10–17 February 2011, with 199 turtles recorded (S2 Table). The combined February 2011 events resulted in the hypothermic stunning of 1,367 turtles or 40% of the 3,381 hypothermic stunned turtles in this environmental parameter analysis. The hypothermic stunned turtles were recorded in the Upper Laguna Madre (n = 475), Lower Laguna Madre (n = 382), Brazos Santiago Pass (n = 475), and rest of the study area (n = 35) during the combined events (Fig 5). The vast majority of turtles were recorded in these three areas during the combined events (98%), and during the other 30 events in the study period (92%). While only 26% of the turtles were recorded in the Upper Laguna Madre during the first phase of the combined events (1–8 February 2011), 86% of the turtles were recorded in the Upper Laguna Madre during the second phase (10–17 February 2011).

We compared air and water temperatures during the combined events with the timing of hypothermic stunning recordings (Fig 5). During the first event, air temperatures at the nearby Bob Hall Pier station dropped from 18.2°C to -2.9°C in 28 hours and then stayed below 5.0°C for about 3 days before returning to 15.0°C and remaining around that temperature for 2.5 days. At the onset of the second event the air temperature again fell below 0.0°C for 10 hours prior to rebounding to levels similar to conditions prior to passage of the strong cold fronts. The Upper Laguna Madre (Bird Island Basin) and Lower Laguna Madre (South Padre Island Coast Guard) experienced similar water and air temperatures, with the southern end of the Lower Laguna Madre water temperatures influenced by the more direct connection to the Gulf of Mexico through the Brazos Santiago Pass. Changes in air temperature preceded changes in water temperature and drove the variability with water temperature decreases at both ends of the Laguna Madre, which were more pronounced than along the Gulf of Mexico coastal waters. During the first cold event, water temperatures in the Upper Laguna Madre dropped from 20.9°C to 10.0°C as the air temperature decreased to its minimum of -2.9°C (Fig 5). Water temperatures continued to decrease reaching a minimum of 3.7°C 75 hours after the start of the first event. Water temperatures then climbed back to above 16.0°C with increases during the daytime and smaller temperature drops during the nighttime until the second cold event. At the onset of the second event, water temperatures in the Upper Laguna Madre dropped to 6.4°C before climbing back above 25.0°C during the daytime a few days later. Throughout these cold events, water temperatures in the nearby Gulf of Mexico remained warmer, above 8.7°C, but did not climb much above 15.0°C. The mean water temperatures during the combined events (30 January to 17 February 2011) were similar (13.7°C, 12.5°C, and 12.3°C) at the three locations (the Upper Laguna Madre, Lower Laguna Madre, and Gulf of Mexico, respectively).

During the study period, virtually all hypothermic stunned turtles (>99%) were found during 32 cold water events with multiple hypothermic stunnings and durations varying from 1 to 21 days (S2 Table). The mean water temperature of two consecutive cold days at the start of the hypothermic stunning event, typically the first and second or the second and third days of hypothermic stunned turtle recordings, were selected to characterize the intensity of a hypothermic stunning event. Hypothermic stunnings were considered to be part of the same event if separated by two days or less and not separated by substantial changes in mean daily water temperatures. The number of turtles recorded in each of these events ranged between 2 and 1,168. All 10 events with 100 or more hypothermic stunnings took place after 2007. The mean of the two day water temperatures of the 10 events prior to 2007 was 6.7°C, while the mean of the 22 events after 2007 was higher at 10.4°C. We did not attempt to correlate hypothermic stunning event size with water temperature because the size of the green turtle population in Texas increased during the study period (Fig 2). Due to the influence of the increasing green turtle population, environmental variables associated with hypothermic stunning events were not weighted by the number of stranded turtles. Ten events with 10–99 hypothermic stunnings and 12 events with 2–9 hypothermic stunnings were identified. During 24 additional events, only a single turtle was recorded, separated by three or more days from larger hypothermic stunning events, and the mean daily water temperatures during these single turtle events was 12.5°C.

Daily environmental conditions were averaged over the 345 hypothermic stunning days/stations. The percentage of environmental data availability is indicated in parentheses. Mean air (52%) and water temperatures (72%) were 10.0°C and 11.4°C respectively. Mean wind speed (74%) was 5.4 m/s and wind gust (70%) was 6.7 m/s. Wind direction (77%) was variable as hypothermic stunning recordings took place over several days including after the strong northerly winds. At the beginning of cold fronts, wind direction was from the north but as cold fronts waned, wind speed decreased and wind direction shifted progressively to the south. Mean barometric pressure (60%) was 1,024 mbar and mean water level (76%) was 0.07 m above station average water level.

To improve environmental data availability, hypothermic stunning events were also associated with conditions at the Upper Laguna Madre station of Bird Island Basin, regardless of location (Table 4). Environmental data availability increased substantially for most variables including, water levels (89%), wave proxy (85%), water temperature (84%), wind speeds (82%), wind gusts (82%), wind directions (84%), and barometric pressures (59%); however air temperature data availability decreased to 37%. As mentioned, air and water temperatures and barometric pressure measured at other locations in the Laguna Madre have correlation strengths of 0.90 or higher with Bird Island Basin data. We compared mean values of environmental parameters during hypothermic stunning events with their related means and standard deviations computed over the winter (November–March). The means of the daily water (11.3°C) and air (11.2°C) temperatures during hypothermic stunning events are lower than their winter means of 17.4°C and 16.8°C minus their respective standard deviations of 4.5°C and 5.1°C. The mean hypothermic stunning day’s air temperature of 11.3°C computed at Bird Island Basin is higher than the mean air temperature of 10.0°C computed over multiple locations, but both data sets are incomplete and air temperature is more variable than water temperature. The hypothermic stunning days water temperatures computed following the two methods are very similar at 11.3°C (Bird Island Basin) and 11.4°C (all stations). Other mean hypothermic stunning day variables computed by the two methods are very similar when taking into account the standard deviation of each variable. Mean values of variables other than air and water temperatures are within the range of the standard deviations around the winter means. The means of the maximum hypothermic stunning day water and air temperatures are both below the winter means of these variables while the mean maxima and minima of the other variables are within or very close to their winter means +/- standard deviation.

**Table 4:** Daily mean environmental conditions recorded at the Bird Island Basin Station during events compared with mean values computed for the overall winter (November–March). The standard deviation is indicated in parenthesis and indicates that only air and water temperatures are substantially different than their winter range during turtle hypothermic stunning events.

Environmental Variable	N	Winter Mean (Standard Deviation)	Variable Mean During Events	Mean of Event Day Maxima	Mean of Event Day Minima	Mean of Event Daily Ranges
Water Level	195	0.18 (0.12)	0.16	0.19	0.13	0.07
Wave Proxy	188	0.002 (0.004)	0.002	0.01	0.0003	0.006
Air Temperature	81	16.8 (5.1)	11.2	15.2	8.5	6.7
Water Temperature	184	17.4 (4.5)	11.3	13.2	10.2	2.9
Wind Speed	181	5.5 (3.0)	5.3	8.6	2.2	6.4
Wind Gusts	181	7.0 (3.6)	6.5	10.4	2.9	7.5
Wind Direction	184	143 (99)	143	281	40	241
Barometric Pressure	130	1019 (7)	1023	1026	1019	7

**Fig 6:**
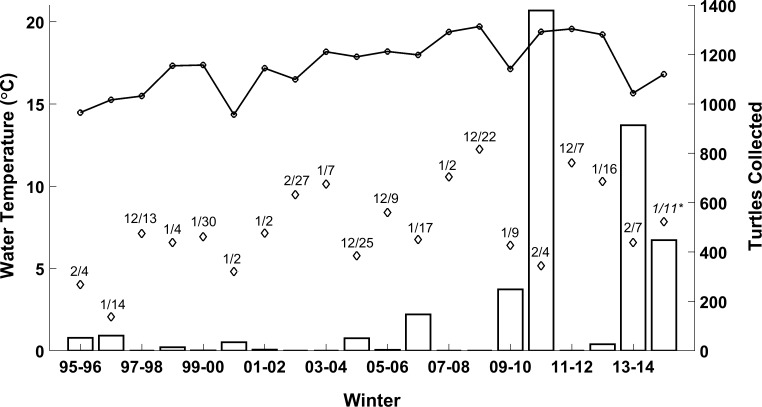
Comparison of the seasonal recording of hypothermic stunned turtles (bars) with winter (November–March) mean water temperatures (full line) and mean water temperature during the two consecutive coldest days of the winters with starting date indicated (diamonds). Water temperatures are for the Upper Laguna Madre (Bird Island Basin). An approximate threshold of 8.0°C identifies winters with substantial numbers of hypothermic stunned turtles recorded. *Water temperatures were not available for the full 2014–2015 winter.

**Fig 7:**
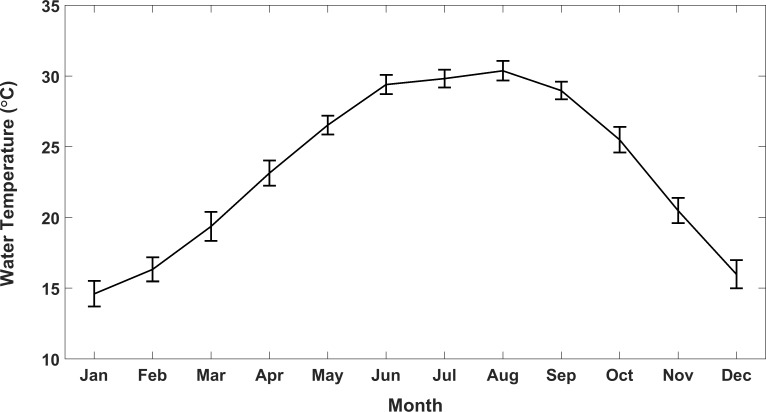
Monthly mean water temperatures for the Upper Laguna Madre computed over the years 1996 through 2014. Statistics computed over the TCOON station of Bird Island Basin. The error bars represent 95% confidence intervals for the monthly means.

The relationship between hypothermic stunning events and water temperatures was further explored by comparing mean yearly winter water temperatures in the Upper Laguna Madre, mean water temperatures of the two consecutive coldest days of each winter, and the number of hypothermic stunned turtles per winter (Fig 6). Mean water temperatures during the winter vary substantially from year to year. The lowest mean winter water temperatures during the study period were 14.5°C (1995–1996), 14.4°C (2000–2001), 15.3°C (1996–1997), and 15.7°C (2013–2014) (Fig 6). The warmest winter water temperatures for the study period were experienced in 2007–2008, 2008–2009, 2010–2011, 2011–2012 and 2012–2013 winters with mean water temperatures between 19.2°C and 19.7°C. As expected these temperatures are substantially lower than the mean full year water temperature of 23.4°C during the study period. Monthly mean water temperatures in the Upper Laguna Madre have a low of 14.6°C in January and a high of 30.4°C in August. The lowest mean temperatures were recorded during the months of November (20.5°C), December (16.0°C), January (14.6°C), February (16.3°C) and March (19.4°C), the same months during which hypothermic stunned turtles have been historically recorded (Fig 7). An approximate threshold of 8.0°C water temperature was found to identify winters with substantial numbers of turtles affected by hypothermic stunning.

Seasonally detrended monthly mean water temperatures in the Upper Laguna Madre were compared with the ENSO, AMO and PDO sea surface temperature pattern indexes (S1 Fig). When considering the year-round time series, water temperatures were only weakly correlated with the ENSO index at the 90% confidence interval (rho = -0.12, p = 0.06), but were significantly correlated with both the PDO (rho = -0.34, p = 0.00) and AMO (rho = 0.23, p = 0.00) indexes. Introducing a lag between the ENSO index and detrended water temperatures resulted in a rapid weakening of the correlation strength. A lag greater than 4 months rapidly diminished the PDO correlation strength as well. When lagging the AMO index, the correlation strength decreased to about 0.18 for an 18 month lag and increased to 0.29 for a 30 month lag. When focusing on winter months only, the correlation strength with the ENSO index (rho = -0.19, p = 0.06) and AMO index (rho = 0.22, p = 0.03) were essentially unchanged compared to year-round correlation strengths; however, the PDO index correlation strength increased from rho = -0.34 to rho = -0.42 (p = 0.00 for both).

## Discussion

Hypothermic stunning occurs in Texas every few years and is the most significant cause of green turtle strandings there, accounting for more strandings than all other sources (e.g., boat strike injuries, debris entanglement, disease) combined. Hundreds to more than 1,600 turtles were found hypothermic stunned per winter during four recent winters, and hence it is expected that large numbers of green turtles will continue to be affected by hypothermic stunning in the future. Targeted search and rescue efforts based on knowledge of the spatial and temporal trends of hypothermic stunning and environmental conditions that cause it will help save the lives of many turtles and facilitate the recovery of this green turtle stock which possesses connectivity across the international border.

### Spatial and temporal trends

The numbers of juvenile green turtles inhabiting Texas waters have increased substantially in recent decades. During the 1950s through the early 1980s, it was thought that there was a low abundance of green turtles in Texas [9,29]. Smith [26] stated that the green turtle was no longer common in the western or northern part of the Gulf of Mexico and Breuer [76] failed to even mention the presence of green turtles in the Lower Laguna Madre during his ecological survey of the area. Rabalais and Rabalais [77] only recorded 10 green turtles among the 259 sea turtles documented stranded in South Texas from September 1976 through September 1979. From the time that the STSSN was established in 1980, through 2015, the annual numbers of green turtles documented stranded in Texas increased exponentially. This increase likely reflects improvements in STSSN coverage [58] and improved public awareness and reporting of strandings. However, it also reflects an increase in juvenile green turtle abundance due to increased recruitment from their likely natal beaches of origin on the Gulf of Mexico coast in Mexico and to a lesser extent in the Caribbean and Florida [59,78], where nesting has surged during the last two decades [37,79–82]. During recent years, other studies have also documented an increase in juvenile green turtles in developmental habitats in the Lower Laguna Madre [31] and along the Atlantic and Gulf coasts of Florida [20,24,83,84].

The largest numbers of green turtle strandings were recorded in Texas during recent years, when the most hypothermic stunned individuals were found and most hypothermic stunned turtles were found in South Texas, particularly the Upper and Lower Laguna Madre. However, it should be cautioned that because some stranded turtles go undetected, particularly in more remote areas, the numbers reported by the STSSN are considered to be minimum estimates of the total number of strandings [63]. Hypothermic stunning occurs mostly inshore, including in remote areas that are sparsely visited by the public and difficult to access by foot or boat. Additionally, the numbers of dead turtles reported by the STSSN likely underestimate at-sea mortality, since only a portion of dead turtles actually wash ashore [85]. Passive transport of dead or injured turtles, before they washed ashore, also may have influenced the distribution that we found for hypothermic stunned stranded turtles; turtles affected by hypothermic stunning in and near the passes may have drifted into the Gulf of Mexico and then washed ashore on Gulf beaches.

### Conditions leading to hypothermic stunning and conservation considerations

Hypothermic stunning can result in substantial mortality, but this mortality can be reduced by human intervention. Prompt detection of these hypothermic stunned turtles has been critical to their survival in Texas. Those found quickly have a better chance of survival than those found days after initiation of a hypothermic stunning event [7]. Larger turtles have more thermal inertia [86] and are among the last to be found affected during hypothermic stunning events. Although some turtles found had other medical conditions that may have predisposed them to hypothermic stunning such as fibropapillomatosis (FP) [87], most of the turtles found appeared to be otherwise healthy. The winter of 2009–2010 was the first since the STSSN was established when more than 450 hypothermic stunned turtles were recorded in Texas during one winter. Only 35.8% of the hypothermic stunned turtles recorded that winter were located alive. However, the percent found alive was much higher during the three subsequent winters when 450 or more hypothermic stunned turtles were recorded in Texas (Table 1), as procedures for detection improved. These enhancements included more rapid searches by STSSN participants and agency personnel, repeated searching of areas where hypothermic stunned turtles had been found, and increased public education to aid with reporting.

Conservation benefits versus financial costs for rescuing and rehabilitating hypothermic stunned and other stranded sea turtles have been questioned in recent years [25,67,88]. The 92.0% rehabilitation success rate for hypothermic stunned green turtles located alive in Texas during the winters of 2006–2007 through 2014–2015 is remarkably high, and only 2.9% (n = 75) of the 2,592 released were subsequently recorded stranded due to hypothermic stunning or another cause, supporting the conservation value of these efforts. During the first decades of the STSSN, when relatively few hypothermic stunned turtles were found during a winter, these turtles were held for months and released after bay waters warmed and the hypothermic stunning season passed. However, in recent years, when more than 450 hypothermic stunned turtles were found during a winter, there was insufficient rehabilitation capacity to enable long-term holding and, under the direction of permitting officials, most of the recuperated turtles were released in the Gulf of Mexico surf off South Texas as soon as water temperatures were above 10°C. These adaptive holding and release strategies, based on numbers of turtles found, holding capacity, and environmental conditions, proved to be successful. Less than 1% of the 2,592 turtles released during the winters of 2006–2007 through 2014–2015 were recaptured within the same winter. Additionally, based on tag returns, most released in Gulf surf waters were able to repatriate back into the bay systems.

Nearly all of our recaptures were in Texas, but one was in Veracruz, Mexico. Additional tag returns and satellite tracking data from stranded and netted green turtles demonstrate regular movement of juvenile green turtles between Texas and the Gulf coast of Mexico [11,30,59], and to our knowledge, hypothermic stunned turtles that were found, rehabilitated, and released in Texas have not been subsequently documented stranded anywhere other than Texas and Mexico through 2015. However, Foley et al. [20] reported that a hypothermic stunned sea turtle found in Florida was recaptured in a U.S. Army Corps of Engineers relocation trawler operating offshore of South Padre Island, Texas 5 years later. None of the hypothermic stunned turtles recorded in Texas have later been documented nesting at any location, although a green turtle captured and tagged in the Lower Laguna Madre during 1991 was recorded nesting in Cuba during 2003 [31]. Insufficient time may have past for most of the hypothermic stunned turtles rehabilitated and released in Texas to have matured and nested since most were small juveniles that were found and released during recent years. However, some may have matured and nested but were not recorded nesting; most of the juveniles inhabiting Texas waters were likely hatched on Gulf coast beaches in Mexico [59] and tag detection efforts are currently minimal for green turtles nesting there. Long-term efforts to document tag returns from these turtles among stranding, in-water capture, and nesting beach surveys is necessary to further quantify the success rate of rehabilitation and contribution of the repatriated turtles to population recovery [66,67].

As winter approaches and water temperatures drop, many sea turtles emigrate from temperate areas and migrate towards warmer tropical waters [7], and as water temperatures decrease in Texas, some juvenile green turtles move in search of warmer waters located further offshore or south. During the winter, Gulf of Mexico waters off Mexico are warmer than those off South Texas [89]. Some green turtles leave South Texas during the winter, while others overwinter there [11,36,60,62]. Shaver et al. [30] found that movements of 30 juvenile green turtles tracked in Texas varied during the winters of 1995–1996, 1996–1997, and 1997–1998; however, most monitored turtles exited the Mansfield Channel during the winter and some later returned following the winter months. Upon exiting the channel, turtles either entered the Lower Laguna Madre or Gulf of Mexico waters off Texas and northern Mexico. Notably, all 7 turtles monitored during the 1996–1997 winter exited the Mansfield Channel area around 12 January 1997 and migrated 60 km south of the U.S.-Mexico border along the Gulf coast of Mexico. This movement was indeed correlated with the passage of a very strong cold front, which was the coldest of that winter so far. Water temperatures declined to 3.7°C on 13 January 1997, which was about 3°C colder than the previous front with coldest temperature (7.1°C) reached on 19 December 1996.

Green turtles that remain in the Laguna Madre during the winter may be particularly susceptible to hypothermic stunning because the shallow water there cools rapidly and there are only three passes to the Gulf of Mexico, where warmer waters can be found. When waters cool rapidly, there may be insufficient time for the turtles to reach deeper channels of the Laguna Madre where waters are warmer or locate one of the three passes to the Gulf of Mexico, thus trapping them where they can fall victim to hypothermic stunning [30]. Similarly, when shallow waters of the IRLS, located on the Atlantic coast of Florida, and of St. Joseph Bay, located on the Gulf coast of Florida, cooled rapidly and dropped below 10°C, resident sea turtles became trapped and hypothermic stunned [4,7,15,16,20,23,90,91]. During major hypothermic stunning events, a large portion of the green turtle population inhabiting the IRLS may be affected [7]. Hypothermic stunning is also a significant threat to sea turtles inhabiting waters of Long Island Sound, Chesapeake Bay, Cape Cod Bay, and other bay systems on the U.S. Atlantic coast during severely cold winter months [17–19,25]. Common geographical characteristics of these areas include relatively shallow bay waters that are semi-enclosed by barrier islands or long peninsulas, with limited openings to access deeper, warmer waters of the Atlantic Ocean or Gulf of Mexico, which compounds the trapping effect.

Climatic and oceanographic factors such as air temperature, sea surface temperature, barometric pressure, wind direction, and wind speed are also known to influence hypothermic stunning events [19,23]. Cold fronts with low barometric pressure and strong winds can bring freezing air temperatures, which in turn lower water temperatures. Weather patterns at a continental scale have been identified as contributing to hypothermic stunning events. Roberts et al. [23] noted that events in Florida generally occur when a negative Arctic Oscillation pattern (-5 to -6) is associated with a mid-level (500 hPa) high amplitude blocking pattern ridge over the western U.S. and trough over the eastern U.S. This results in frigid Arctic air masses in lower latitudes accompanied by strong, northwesterly prevailing winds. These winds influence the aggregation of hypothermic stunned turtles on windward-facing shorelines, and the greater the amount of time that water temperatures remain below 10°C (>12–24 hours) also affects the magnitude of stunned turtles [23].

Environmental conditions resulting in turtle hypothermic stunnings in the Laguna Madre were generally similar to conditions in other locations in the U.S. for which there are data. Mean air and water temperatures clearly stand out with event means lower than winter means minus standard deviations (Table 4). Water temperatures can change rapidly in the Laguna Madre, driven by the cooling air temperature of a cold front and a low thermal inertia. More than 99% of the study period’s hypothermic stunned turtles were recorded during or shortly after the passage of strong cold fronts, rightfully focusing the attention of preparation and planning around these events and their likelihood.

For this study, mean and standard deviation values of other environmental variables monitored over hypothermic stunned turtle recording days (wind, barometric pressure, and water level) were all within their winter means. This result is in part due to averaging over the several days during which hypothermic stunned turtles are documented. A total of 32 multi turtle events were identified with the mean length of an event at 7.5 days and a maximum event duration of 21 days. While winds are stronger than average over the first one to three days of a front, winds thereafter decrease and turn southerly during the following days while hypothermic stunned turtles are still recorded. For the longer events, additional cold fronts can prolong the event duration. Weighing environmental conditions by the number of recorded turtles would have resulted in different means. Additionally, averaging environmental values over the first two or three days of the events would have also resulted in different means, including stronger and more northerly winds.

Establishing a threshold water temperature for the occurrence of hypothermic stunning events is important for response planning and preparation. Mean water temperatures during days when hypothermic stunned turtles were recorded were 11.4°C when averaging over locations nearest to the recorded hypothermic stunning locations and 11.3°C based on the more complete but often more distant Upper Laguna Madre data set. Basing a threshold on the day of the documentation overestimates the threshold temperature as some turtles will drift and not be found and recorded until a day or longer after they become incapacitated. Furthermore, since individual cold fronts take place in the context of preceding events, other cold fronts and the winter’s overall water temperature history will affect turtles. For example, the number of recorded hypothermic stunned turtles was higher in February (49%) than in January (35%), yet the mean water temperature was higher in February (16.3°C) than in January (14.6°C).

For establishing a threshold water temperature, mean winter water temperatures (Fig 6) are not a good predictor of hypothermic stunning events either. Strong cold fronts with low air temperatures are short term meteorological events, they can take place as part of a series of such events during a cold winter but they can also take place during an otherwise mild winter. An example is the 2010–2011 winter which saw the largest number of hypothermic stunned turtles yet that winter and was one of the mildest of the study period, being one of the five winters with mean water temperatures above 19.2°C. In comparison, several winters had considerably lower mean water temperatures, with five below 15.7°C.

Another method to establish a threshold water temperature is based on seasonal statistics from the environmental parameter analysis, specifically the total number of hypothermic stunned turtles and the mean water temperature of the two consecutive coldest days of the winter (Fig 6). The latter characterizes the cold front with the lowest temperatures of the winter. A winter was defined as significant when 15 or more hypothermic stunned turtles were recorded. The first two winters of the data set, 1995–1996 (52 turtles) and 1996–1997 (61 turtles), are part of this group indicating that the threshold is not overly sensitive to the growth of the turtle population. The breakdown results in two sets of 10 winters with respective two-day consecutive coldest water temperature means of 6.0°C and 9.0°C. This suggests a water temperature threshold around 7.5°C. Further consideration of individual events points to the 2014–2015 winter with a mean water temperature for the two coldest consecutive days of 7.9°C and a total of 448 hypothermic stunned turtles. Hypothermic stunned turtle rescues took place mainly as part of two events starting with a 13 November 2014 event (145 turtles) and followed later that season by a longer event starting 3 January 2015 (292 turtles). Water temperatures were not available for the TCOON station of Bird Island Basin during the second event, potentially missing lower water temperatures. Overall, none of the winters with two-day mean water temperatures higher than 8.0°C had numbers of hypothermic stunned turtles larger than 15, except for the 2012–2013 winter with 26 turtles. During that season, the largest hypothermic stunning event started on 15 January 2013 and totaled 13 turtles over seven days. While the lowest two-day mean water temperature for this winter was 10.3°C, the threshold mean water temperature was not raised as the number of hypothermic stunned turtles for that winter was barely above the initially set threshold of 15 turtles and 2012–2013 is one of the last seasons of the study period and hence was influenced by the growth of the green turtle population. Based on the results of this study and the latter methodology, a threshold mean temperature of 8.0°C is suggested to predict large turtle hypothermic stunning events.

During the largest hypothermic stunning event of 1,168 turtles, shortly followed by another large event of 199 turtles, hypothermic stunnings took place in the Upper Laguna Madre (n = 473), Lower Laguna Madre (n = 382) and the Brazos Santiago Pass (n = 475), in numbers consistent with the relative proportions of turtles from these areas over the course of this study. However, while only 26% of the turtles were recorded in the Upper Laguna Madre during the first part of this period (1–8 February 2011), 86% of the turtles were recorded in the Upper Laguna Madre during the second phase (10–17 February 2011). This difference could indicate that turtles in the southern end of the Laguna Madre were able to transit to the Gulf of Mexico helped by the strong northerly winds and wind generated currents, while turtles in the northern end of the Laguna Madre, pushed southward by winds, may have had difficulties exiting the Laguna Madre through the Packery Channel, located at the northern end of the Upper Laguna Madre (Fig 1).

Correlations of Laguna Madre water temperatures with broad sea surface temperature pattern fluctuations have the potential to guide preparation efforts ahead of a winter. Overall, the strongest relationship between water temperatures and the climatic indexes tested was found for the PDO with correlation strengths of rho = -0.42 for the winter months and rho = -0.34 for year-round temperatures (p = 0.00 for both). These results should be taken with caution given the periodicity of the PDO, and the multi-decadal occurrence of the AMO, which are considerably longer than the study period. This study shows that the strong cold events in the Laguna Madre over the past two decades that generated significant hypothermic stunning did not necessarily take place during the coldest winters of this time frame (Fig 6). Nevertheless, the significant correlations between water temperatures in the Laguna Madre and the fluctuations of the PDO and AMO indexes could be further investigated with increases in the PDO index correlating with lower mean water temperatures, and increases in the AMO index correlating with higher mean water temperatures.

## Conclusion

To continue to help recover this regionally distinct population, comply with U.S. Endangered Species requirements, and respond to humanitarian concerns, federal and state permitting entities direct that STSSN participants locate hypothermic stunned turtles quickly to help ensure as many survive as possible, minimize holding time to that medically necessary, and release surviving individuals into Gulf of Mexico surf waters as soon as these waters warm, or back into bays if that winter’s hypothermic stunning season is over. Without rescue, rehabilitation, and repatriation of these hypothermic stunning victims, the green turtle population in Texas would be smaller. Attempts should be made to locate hypothermic stunned turtles as soon as water temperatures fall below 10°C. A threshold mean water temperature of 8.0°C computed over the two consecutive coldest days of a winter was found to identify winters with none or few hypothermic stunnings (higher than 8.0°C) and winters with larger hypothermic stunning events (lower than 8.0°C). Tools to accurately predict water temperatures approximately 48 hours in advance allow responders to prepare resources for turtle recoveries and redirect activities such as navigation and coastal engineering work away from identified target areas where hypothermic turtles are likely to be found.

## Supporting information

S1 Table Cross correlations between environmental parameters measured at the Bird Island Basin station and seven other TCOON/NWLON monitoring stations throughout the Laguna Madre in south Texas from the winters of 1995–1996 through 2014–2015 (all p-values at 0; no lags included). Associations computed based on available measurements with time series spans varying depending on station location and environmental parameter. Pearson correlation coefficients were used for non-directional variables and circular-circular correlation coefficients [92] were used for wind direction correlations.
(PDF)

S2 Table Summary of stranded turtles with date, duration of the event, and mean water temperature of the initial two consecutive cold days of the events in the environmental parameter analysis. (PDF)

S1 Fig Comparison of seasonally detrended monthly mean water temperatures in the Upper Laguna Madre with three seasonal indexes ENSO (red), AMO (blue) and PDO (green). (TIF)

## References

[1] George RH. Health problems and diseases of sea turtles In: Lutz PL, Musick JA, editors. The biology of sea turtles. Boca Raton: CRC Press; 1997 pp. 363–385.

[2] Spotila JR, O'Connor MP, Paladino FV. 1997 Thermal biology. In: Lutz PL, Musick JA, editors. The biology of sea turtles. Boca Raton: CRC Press; 1997. pp. 297–314.

[3] Davenport J. Temperature and the life-history strategies of sea turtles. J Therm Biol. 1997; 22: 479–488.

[4] Mendonça MT. Movements and feeding ecology of immature green turtles (*Chelonia mydas*) in a Florida lagoon. Copeia. 1983: 1013–1023.

[5] Spotlia JR, Standora JR. Environmental constraints on the thermal energetics of sea turtles. Copeia. 1985: 694–702.

[6] Schwartz FJ. Behavioral and tolerance responses to cold water temperatures by three species of sea turtles (*Reptilia*, *Chelonidae*) in North Carolina. Fla Mar Res Publ. 1978; 33: 16–18.

[7] Witherington BE, Ehrhart LE. Hypothermic stunning and mortality of marine turtles in the Indian River Lagoon system, Florida. Copeia. 1989; 3: 696–703.

[8] Ogren L, McVea C. Apparent hibernation by sea turtles in N. American waters In: Bjorndal K, editor. Biology and conservation of sea turtles. Washington DC: Smithsonian Institution Press; 1982 pp. 127–132.

[9] Hildebrand HH. A historical review of the sea turtle populations in the Western Gulf of Mexico In: Bjorndal K, editor. Biology and conservation of sea turtles. Washington DC: Smithsonian Institution Press; 1982 pp. 447–453.

[10] Doughty RW. Sea turtles in Texas: a forgotten commerce. Southwest Hist Q. 1984; 88: 43–69.

[11] Shaver DJ. Distribution, residency, and seasonal movements of the green sea turtle, Chelonia mydas (Linnaeus, 1758) in Texas. Ph.D. Dissertation, Texas A&M University. 2000.

[12] Brongersma LD. Marine turtles of the eastern Atlantic Ocean In: Bjorndal K, editor. Biology and conservation of sea turtles. Washington DC: Smithsonian Institution Press; 1982 pp. 407–416.

[13] Brill RW, Balazs GH, Holland KN, Chang RKC, Sullivan S, George JC. Daily movements, habitat use, and submergence intervals of normal and tumor-bearing juvenile green turtles (*Chelonia mydas* L.) within a foraging area in the Hawaiian Islands. J Exp Mar Bio Ecol. 1995; 185: 203–218.

[14] Shoop C. Sea turtles in the northeast. Maritimes. 1980; 24: 9–11.

[15] Mendonça MT. Comparative growth rates of wild immature *Chelonia mydas* and *Caretta caretta* in Florida. J Herpetol. 1981; 15: 444–447.

[16] Mendonça MT, Ehrhart LM. Activity, population size and structure of immature *Chelonia mydas* and *Caretta caretta* in Mosquito Lagoon, Florida. Copeia. 1982; 1: 161–167.

[17] Burke VJ, Standora EA. Factors affecting stranding of cold-stunned juvenile Kemp's ridley and loggerhead sea turtles in Long Island, New York. Copeia. 1991; 4: 1136–1138.

[18] Morreale SJ, Meylan AB, Sadove SS, Standora EA. Annual occurrence and winter mortality of marine turtles in New York waters. J Herpetol. 1992; 26: 301–308.

[19] Still BM, Griffin CR, Prescott R. Climatic and oceanographic factors affecting daily patterns of juvenile sea turtle cold-stunning in Cape Cod Bay, Massachusetts. Chelonian Conserv Biol. 2005; 4: 883–890.

[20] Foley AM, Singel K, Dutton PH, Summer T, Redlow AE, Lessman J. Characteristics of a green turtle (*Chelonia mydas*) assemblage in northwestern Florida determined during a hypothermic stunning event. Gulf Mex. Sci. 2007: 131–143.

[21] Anderson ET, Harms CA, Stringer EM, Cluse WM. Evaluation of hematology and serum biochemistry of cold-stunned green sea turtles (*Chelonia mydas*) in North Carolina, USA. J Zoo Wildl Med. 2011; 42: 247–255. 10.1638/2010-0217.1 22946402

[22] Williams NC, Bjorndal KA, Lamont MM, Carthy RR. Winter diets of immature green turtles (*Chelonia mydas*) on a northern feeding ground: integrating stomach contents and stable isotope analyses. Estuaries Coast. 2013; 37: 986–994.

[23] Roberts K, Collins J, Paxton CH, Hardy R, Downs J. Weather patterns associated with green turtle hypothermic stunning events in St. Joseph Bay and Mosquito Lagoon, Florida. Phys Geogr. 2014; 35: 134–150.

[24] Avens L, Goshe LR, Harms CA, Anderson ET, Goodman Hall A, Cluse WM, et al Population characteristics, age structure, and growth dynamics of neritic juvenile green turtles in the northeastern Gulf of Mexico. Mar Ecol Prog Ser. 2012; 458: 213–229.

[25] Godfrey M. Turtles left out in the cold? Current Conserv. 2013; 6 2:31–33.

[26] Smith FGW. Taxonomy and distribution of sea turtles. In: Galtsoff PS, editor. Gulf of Mexico: its origin, waters, and marine life. Washington DC: Fishery Bulletin 89; 1954. pp. 513–515.

[27] Witzell WN. The origin, evolution, and demise of the U.S. sea turtle fisheries. Mar Fish Rev. 1994; 56: 8–23.

[28] Witzell WN. The U.S. commercial sea turtle landings. NOAA Tech. Memo. NMFS-SEFSC-350. Miami: U.S. Department of Commerce; 1994.

[29] Hildebrand HH. Random notes on sea turtles in the Western Gulf of Mexico. In: Owens D, Crowell D, Dienberg G, Grassman M, McCain S, Morris Y, et al., editors. Western Gulf of Mexico sea turtle workshop proceedings. TAMU-SG-84-105. College Station: Sea Grant College Program, Texas A&M University; 1983. pp. 34–40.

[30] Shaver DJ, Hart KM, Fujisaki I, Rubio C, Sartain AR. Movement mysteries unveiled: spatial ecology of juvenile green sea turtles In: Lutterschmidt WI, editor. Reptiles in research. Hauppauge: Nova Science Publishers, Inc.; 2013 pp. 463–483.

[31] Metz TL, Landry AM Jr. An assessment of green turtle (*Chelonia mydas*) stocks along the Texas coast, with emphasis on the Lower Laguna Madre. Chelonian Conserv Biol. 2013; 12: 293–302.

[32] Hirth HF. Synopsis of the biological data on the green turtle *Chelonia mydas* (Linnaeus 1758). Washington DC: U.S. Department of the Interior, U.S. Fish and Wildlife Service; 1997.

[33] Musick JA, Limpus CJ. Habitat utilization and migration in juvenile sea turtles In: Lutz PL, Musick JA, editors. The biology of sea turtles. Boca Raton: CRC Press; 1997 pp. 137–163.

[34] Witherington BE, Hirama S, Hardy R. Young sea turtles of the pelagic *Sargassum*-dominated drift community: habitat use, population density, and threats. Mar Ecol Prog Ser. 2012; 463: 1–22.

[35] Collazo JA, Boulon R, Tallevast TL. Abundance and growth patterns of *Chelonia mydas* in Culebra, Puerto Rico. J Herpetol. 1992; 26: 293–300.

[36] Shaver DJ. Relative abundance, temporal patterns, and growth of sea turtles at the Mansfield Channel, Texas. J Herpetol. 1994; 28: 491–497.

[37] Meylan PA, Meylan AB, Gray JA. The ecology and migrations of sea turtles 8. Tests of the developmental habitat hypothesis. Bull Am Mus of Nat His. 2011; 357: 70.

[38] Bjorndal KA. Nutrition and grazing behavior of the green turtle, *Chelonia mydas*. Mar Biol. 1980; 56: 147–154.

[39] Bjorndal KA. The consequences of herbivory for the life history pattern of the Caribbean green turtle In: Bjorndal K, editor. Biology and conservation of sea turtles. Washington DC: Smithsonian Instituition Press; 1982 pp. 111–116.

[40] Bjorndal KA. Foraging ecology and nutrition of sea turtles In: Lutz PL, Musick JA, editors. The biology of sea turtles. Boca Raton: CRC Press; 1997 pp. 199–232.

[41] Howell LN, Reich KJ, Shaver DJ, Landry AM Jr, Gorga CC. Ontogenetic shifts in diet and habitat of juvenile green sea turtles in the northwestern Gulf of Mexico. Mar Ecol Prog Ser. 2016; 559: 217–229.

[42] Velez-Zuazo X, Quinones J, Pacheco AS, Klinge L, Paredes E, Quispe S, et al Fast growing, healthy and resident green turtles (*Chelonia mydas*) at two neritic sites in the central and northern coast of Peru: implications for conservation. PloS ONE. 2014; 9(11):e113068 10.1371/journal.pone.0113068 25409240 PMC4237379

[43] Sampson L, Giraldo A, Payan LF, Amorocho DF, Eguchi T, Seminoff JA. Somatic growth of juvenile green turtle (*Chelonia mydas*) morphotypes in the Colombian Pacific. Mar Biol. 2015.

[44] Balazs GH. Green turtle migrations in the Hawaiian Archipelago. Biol Conserv. 1976; 9: 125–140.

[45] Blanco GS, Morreale SJ, Bailey H, Seminoff JA, Paladino FV, Spotila JR. Post-nesting movements and feeding grounds of a resident East Pacific green turtle *Chelonia mydas* population from Costa Rica. Endanger Species Res. 2012; 18: 233–245.

[46] Baudouin M, De Thoisy B, Chambault P, Berzins R, Entraygues M, Kelle L, et al Identification of key marine areas for conservation based on satellite tracking of post-nesting migrating green turtles (*Chelonia mydas*). Biol Conserv. 2015; 184: 36–41.

[47] Shew DM, Baumann RH, Fritts TH, Dunn LS. Texas barrier islands region ecological characterization: environmental synthesis papers. FWS/OBS-81/32. Washington DC: U.S. Department of the Interior, U.S. Fish and Wildlife Service; 1981.

[48] Pulich Jr W, Onuf C. Statewide summary for Texas. In: Handley L, Altsman D, De May R, editors. Seagrass status and trends in the northern Gulf of Mexico: 1940–2002. U.S. Geological Survey Scientific Investigations Report 2006–1587 and U.S. Environmental Protection Agency 855-R-04-003; 2007. pp. 7–17.

[49] Quammen ML, Onuf CP. Laguna Madre: seagrass changes continue decades after salinity reduction. Estuaries. 1993; 16: 302–310.

[50] Tunnell JW. Geography, climate, and hydrography In: Tunnell JW, Judd FW, editors. The Laguna Madre of Texas and Tamaulipas. College Station: Texas A & M University Press; 2002 pp. 7–27.

[51] Withers K. Red and brown tides In: Tunnell JW, Judd FW, editors. The Laguna Madre of Texas and Tamaulipas. College Station: Texas A & M University Press; 2002 pp. 255–258.

[52] Hicks DW, Tunnell JW. Ecological notes and patterns of dispersal in the recently introduced mussel, *Perna perna* (Linne, 1758), in the Gulf of Mexico. Am Malacol Bull. 1995; 11: 203–206.

[53] Kaldy JE, Dunton KH, Czerny AB. Variation in macroalgal species composition and abundance on a rock jetty in the Northwest Gulf of Mexico. Botanica Marina. 1995; 38: 519–527.

[54] Lehman RL. A checklist of benthic marine macroalgae from the Corpus Christi Bay area. Tex J Sci. 1999; 51: 241–252.

[55] Lehman RL. Marine plants of the Texas coast. College Station: Texas A&M University Press; 2013.

[56] Wynne M. A checklist of benthic marine algae of the coast of Texas. Gulf Mex Sci. 2009; 1: 64–87.

[57] Fikes R, Lehman R. Recruitment and colonization of macroalgae to a newly developed rocky intertidal habitat in the Northwest Gulf of Mexico. Gulf Caribb Res. 2010; 22: 9–20.

[58] Shaver DJ. Sea turtle strandings along the Texas coast, 1980–94. In: Zimmerman R, editor. Characteristics and causes of Texas marine strandings. NOAA Technical Reports NMFS 143. Miami: U.S. Department of Commerce; 1998. pp. 57–72.

[59] Shamblin BM, Dutton PH., Shaver DJ, Bagley DA, Putman NF, Mansfield KL, et al Mexican origins for the Texas green turtle foraging aggregation: a cautionary tale of incomplete baselines and poor marker resolution. J Exp Mar Biol Ecol. 2016.

[60] Coyne MS. Feeding ecology of subadult green sea turtles in South Texas waters. M.Sc. Thesis, Texas A&M University. 1994.

[61] Renaud ML, Carpenter JA, Williams JA, Manzella-Tirpak SA. Activities of juvenile green turtles, *Chelonia mydas*, at a jettied pass in South Texas. Fish Bull. 1995; 93: 586–593.

[62] Arms SA. Overwintering behavior and movement of immature green sea turtles in South Texas waters. M.Sc. Thesis, Texas A&M University. 1996.

[63] Teas WG. Species composition and size class distribution of marine turtle strandings on the Gulf of Mexico and southeast United States coast, 1985–1991. NOAA Tech. Memo. NMFS-SEFSC-315. Miami: U.S. Department of Commerce; 1993.

[64] Magnuson JJ, Bjorndal KA, Dupaul WD, Graham GL, Owens DW, Peterson CH, Pritchard PCH, Richardson JI, Saul GE, West CW. Decline of the sea turtles: causes and prevention. Washington DC: National Research Council, National Academy Press; 1990.

[65] Shaver DJ, Teas WG. Stranding and salvage networks In: Eckert K, Bjorndal KA, Abreu-Grobois FA, Donnelly M, editors. Research and management techniques for the conservation of sea turtles. Washington DC: IUCN/SSC Marine Turtle Specialist Group; 1999 pp. 152–155.

[66] Coleman AT, Pulis EE, Pitchford JL, Crocker K, Heaton AJ, Carron AM et al Population ecology and rehabilitation of incidentally captured Kemp’s ridley sea turtles (*Lepidochelys kempii*) in the Mississippi Sound, USA. Herpetol Conserv Biol. 2016; 11: 253–264.

[67] Caillouet CW Jr, Putman NF, Shaver DJ, Valverde RA, Seney EE, Lohmann KJ, et al A call for evaluation of the contribution made by rescue, resuscitation, rehabilitation, and release translocations to Kemp’s ridley sea turtle (*Lepidochelys kempii*) population recovery. Herpetol Conserv Biol. 2016; 11: 486–496.

[68] NMFS, USFWS. Recovery plan for U.S. population of Atlantic green turtle. Washington DC: U.S. Department of Commerce, National Marine Fisheries Service; 1991.

[69] Bjorndal KA. Priorities for research in foraging habitats In: Eckert KL, Bjorndal KA, Abreu-Grobois FA, Donnelly M, editors. Research and management techniques for the conservation of sea turtles. Washington DC: IUCN/SSC Marine Turtle Specialist Group; 1999 pp. 12–14.

[70] Hamann M, Godfrey MH, Seminoff JA, Arthur KE, Barata PCR, Bjorndal KA, et al Global research priorities for sea turtles: informing management and conservation in the 21st century. Endanger Species Res. 2010; 11: 245–269.

[71] Balazs GH. Factors to consider in the tagging of sea turtles In: Eckert KL, Bjorndal KA, Abreu-Grobois FA, Donnelly M, editors. Research and management techniques for the conservation of sea turtles. Washington DC: IUCN/SSC Marine Turtle Specialist Group; 1999 pp. 101–109.

[72] Murray LS, Jinnette TS. Survival of dominant estuarine organisms impinged on fine mesh traveling screens at the Barney M. Davis Power Station. In: Sharma RK, Palmer JB, editors. Larval exclusion systems for power plant cooling water intakes. Argonne: Argonne National Laboratory, Publication ANVES-66; 1978. pp. 79–87.

[73] Environmental Systems Research Institute (ESRI). ArcGIS Desktop: release 10.2.1; 2014.

[74] Wilks D. Statistical Methods in Atmospheric Sciences. Third Edition Oxford: Elsevier; 2011.

[75] Armstrong NE. The ecology of open-bay bottoms of Texas: a community profile. Washington DC: Biological Reports 85(7.12), US Department of the Interior, Fish and Wildlife Service; 1987.

[76] Breuer JP. An ecological survey of the lower Laguna Madre of Texas, 1953–1959. Pubs Inst Mar Sci. 1962; 8: 153–183.

[77] Rabalais SC, Rabalais NN. The occurrence of sea turtles on the South Texas coast. Contr Mar Sci. 1980; 23: 123–129.

[78] Anderson JD, Shaver DJ, Karel WJ. Genetic diversity and natal origins of green turtles (*Chelonia mydas*) in the western Gulf of Mexico. J Herpetol. 2013; 47(2): 251–257.

[79] Xavier R, Barata A, Cortez LP, Queiroz N, Cuevas E. Hawksbill turtle (*Eretmochelys imbricata* Linnaeus 1766) and green turtle (*Chelonia mydas* Linnaeus 1754) nesting activity (2002–2004) at El Cuyo beach, Mexico beach, Mexico. Amphibia-Reptilia. 2006; 27: 539–547.

[80] Chaloupka MY, Bjorndal KA, Balazs GH, Bolten AB, Ehrhart LM, Limpus CJ, et al Encouraging outlook for recovery of a once severely exploited marine megaherbivore. Glob Ecol Biogeogr. 2008; 17: 297–304.

[81] Zavaleta-Lizarraga L, Morales-Mavil JE. Nest site selection by the green turtle (*Chelonia mydas*) in a beach of the north of Veracruz, Mexico. Rev Mex Biodivers. 2013; 84: 927–937.

[82] Seminoff JA, Allen CD, Balazs GH, Dutton PH, Eguchi T, Haas HL, et al. Status review of the green turtle (Chelonia mydas) under the Endangered Species Act. NOAA-TM-NMFS-SWFSC-539. LaJolla: U.S. Department of Commerce, National Marine Fisheries Service; 2015.

[83] Ehrhart LM, Redfoot WE, Bagley DA. Marine turtles of the central region of the Indian River Lagoon System, Florida. Fla Sci. 2007; 70(4): 415–434.

[84] Redfoot W, Ehrhart L. Trends in size class distribution, recaptures, and abundance of juvenile green turtles (*Chelonia mydas*) using a rock riprap lined embayment at Port Canaveral, Florida, USA, as a developmental habitat. Chelonian Conserv Biol. 2013; 12: 252–261.

[85] Epperly SP, Braun J, Chester AJ, Cross FA, Merriner JV, Tester PA, et al Beach strandings as an indicator of at-sea mortality of sea turtles. Bull Mar Sci. 1996; 59: 289–297.

[86] Sato K. Body temperature stability achieved by the large body mass of sea turtles. J Exp Biol. 2014; 217: 3607–3614. 10.1242/jeb.109470 25147244

[87] Tristan T, Shaver DJ, Kimbro J, deMaar T, Metz T, George J, Amos A. Identification of fibropapillomatosis in green sea turtles (*Chelonia mydas*) on the Texas coast. J Herpetol Med Surg. 2010; 20(4): 109–112.

[88] Baker L, Edwards W, Pike DA. Sea turtle rehabilitation success increases with body size and differs among species. Endanger Species Res. 2015; 29: 13–21.

[89] Inoue M, Welsh SE. Numerical simulation of Gulf of Mexico circulation under present and glacial climatic conditions, OCS Study MMS 96–0067. New Orleans: U.S. Dept. Interior, Minerals Management Service; 1997.

[90] Zug GR, Glor RE. Estimates of age and growth in a population of green sea turtles (*Chelonia mydas*) from the Indian River lagoon system, Florida: a skeletochronological analysis. Can J Zool. 1998; 76: 1497–1506.

[91] Lamont MM, Fujisaki I, Stephens BS, Hackett C. Home range and habitat use of juvenile green turtles (*Chelonia mydas*) in the northern Gulf of Mexico. Anim Biotelemetry. 2015.

[92] Berens P. CircStat: a MATLAB toolbox for circular statistics. J Stat Softw. 2009; 31(10): 1–21.

