# Supplementary material for: Hypothermic stunning of green sea turtles in a western Gulf of Mexico foraging habitat
Source: PLoS One. 2017 Mar 17;12(3):e0173920. doi: 10.1371/journal.pone.0173920 (PMC5357020; doi:10.1371/journal.pone.0173920)
Supplement: S1 Table — Associations computed based on available measurements with time series spans varying depending on station location and environmental parameter. Pearson correlation coefficients were used for non-directional variables and circular-circular correlation coefficients [92] were used for wind direction correlations. (PDF) [file pone.0173920.s001.pdf]

S1 Table. Cross correlations between environmental parameters measured at the Bird Island Basin station and seven other TCOON/NWLON monitoring stations throughout the Laguna Madre in south Texas from the winters of 1995–1996 through 2014–2015 (all p-values at 0; no lags included). Associations computed based on available measurements with time series spans varying depending on station location and environmental parameter. Pearson correlation coefficients were used for non-directional variables and circular-circular correlation coefficients [92] were used for wind direction correlations.

Environmental Parameter	Packery Channel	Bob Hall Pier	Baffin Bay	Rincon del San Jose	Port Mansfield	Realitos	SPI Coast Guard
Water Temperature	0.96	0.94	0.96	0.95	0.96	0.96	0.90
Air Temperature	0.99	0.98	0.99	0.98	0.97	0.97	0.97
Water Level	0.93	0.46	0.96	0.57	0.85	0.69	0.51
Wind Speed	0.86	0.80	0.87	0.76	0.48	0.68	0.48
Wind Gust	0.89	0.81	0.88	0.77	0.57	0.71	0.63
Wind Direction	0.91	0.90	0.87	0.73	0.71	0.77	0.69
Barometric Pressure	1.00	1.00	1.00	0.97	1.00	0.99	0.96
