# Supplementary material for: Hypothermic stunning of green sea turtles in a western Gulf of Mexico foraging habitat
Source: PLoS One. 2017 Mar 17;12(3):e0173920. doi: 10.1371/journal.pone.0173920 (PMC5357020; doi:10.1371/journal.pone.0173920)
Supplement: S2 Table — (PDF) [file pone.0173920.s002.pdf]

S2 Table. Summary of stranded turtles with date, duration of the event, and mean water temperature of the initial two consecutive cold days of the events in the environmental parameter analysis.

Combined Number of Turtles Recorded Per Event	Start Date	Duration of Event (Number of Days)	Consecutive 2-Day Mean Water Temperature (ºC)
1168	2/1/2011	8	5.8
292	1/3/2015	17	NA
240	12/5/2013	13	8.6
239	1/6/2010	12	6.4
229	2/6/2014	9	8.4
199	2/10/2011	8	8.5
198	1/6/2014	12	7.0
181	1/22/2014	13	10.5
146	1/17/2007	21	6.8
145	11/13/2014	13	9.9
58	1/13/1997	11	2.5
50	12/25/2005	9	5.8
48	11/25/2013	6	11.1
44	2/4/1996	4	4.0
15	12/28/2001	9	8.4
14	12/26/1999	14	NA
13	1/15/2013	8	11.2
13	12/28/2014	7	11.1
12	12/14/2000	9	8.4
10	1/12/2011	3	9.7
7	1/5/2013	3	11.5
5	12/26/2012	5	15.3
4	1/2/2002	5	7.1
4	12/5/2009	3	9.3
4	12/4/2000	5	9.2
4	2/24/2015	3	11.2
3	1/19/1996	1	8.3
3	3/6/2015	1	NA
2	12/232013	2	14.7
2	2/23/2014	2	19.5
2	1/25/2015	2	12.3
2	12/26/1995	2	7.7
24	NA	1[1]	NA

[1] 1 Day events with 1 stranded turtle recorded from 1996 through 2014
