# Supplementary figures and images for: Hypothermic stunning of green sea turtles in a western Gulf of Mexico foraging habitat
Source: PLoS One. 2017 Mar 17;12(3):e0173920. doi: 10.1371/journal.pone.0173920 (PMC5357020; doi:10.1371/journal.pone.0173920)

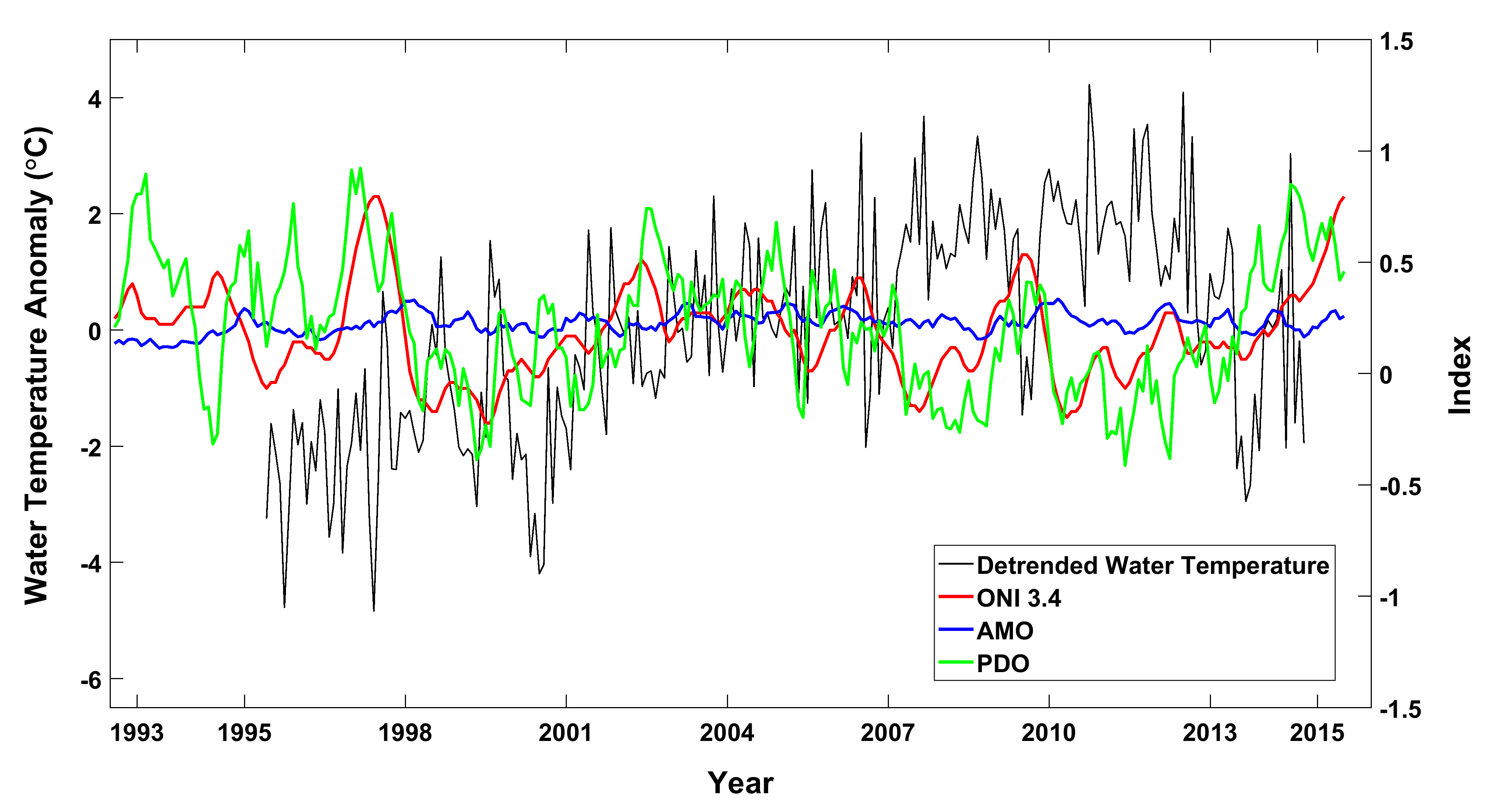

Supplement: S1 Fig — (TIF) [file pone.0173920.s003.tif]
